# RPL17 Promotes Colorectal Cancer Proliferation and Stemness through ERK and NEK2/β-catenin Signaling Pathways

**DOI:** 10.7150/jca.69428

**Published:** 2022-05-13

**Authors:** Min Ji Ko, Yu-Ri Seo, Daekwan Seo, So-Young Park, Ji Hae Seo, Eun-Hye Jeon, Sang-Woo Kim, Keon Uk Park, Deog-Bon Koo, Shin Kim, Jae-Hoon Bae, Dae-Kyu Song, Chi Heum Cho, Kwang Seok Kim, Yun-Han Lee

**Affiliations:** 1 Department of Molecular Medicine, Keimyung University School of Medicine, Daegu 42601, South Korea.; 2 Psomagen Inc., Rockville, MD 20850, USA.; 3 Department of Biochemistry, Keimyung University School of Medicine, Daegu 42601, South Korea.; 4 Department of Biological Sciences, Pusan National University, Pusan 46241, South Korea.; 5 Department of Internal Medicine, Keimyung University School of Medicine, Daegu 42601, South Korea.; 6 Department of Biotechnology, College of Engineering, Daegu University, Gyeongbuk 38453, South Korea.; 7 Department of Immunology, Keimyung University School of Medicine, Daegu 42601, South Korea.; 8 Department of Physiology & Obesity-mediated Disease Research Center, Keimyung University School of Medicine, Daegu 42601, South Korea.; 9 Department of Obstetrics and Gynecology, Keimyung University School of Medicine, Daegu 42601, South Korea.; 10 Division of Radiation Cancer Research, Korea Institute of Radiological & Medical Sciences, Seoul 01812, South Korea.

**Keywords:** colorectal cancer, RPL17, ERK, NEK2, proliferation, stemness

## Abstract

**Aims:** Ribosomal protein L17 (RPL17), a 60S subunit component, is up-regulated in colorectal cancer (CRC). However, its oncogenic role in CRC progression remains unexplored. Thus, we aimed to investigate the effect of RPL17 targeting on CRC *in vitro* and *in vivo* and whether RPL17 gained an extra-ribosomal function during CRC development.

**Methods:** RPL17-specific siRNAs complexed with cationic lipids were transfected to CRC cells to silence target gene expression and then real-time RT-PCR and western blotting were applied to observe the change of expression or activity of genes or proteins of interest. Cell proliferation assay, clonogenic assay and cell cycle analysis were used to determine the in vitro effects of RPL17siRNAs on CRC cell growth, and a subcutaneous xenograft assay was applied to test the effect of RPL17siRNAs on in vivo tumor growth. RNA sequencing and western blotting were used to investigate the underlying mechanisms. Sphere-forming assay, invasion assay and migration assay were used to evaluate the effects of RPL17siRNAs on CRC stemness.

**Results:** siRNA-mediated inhibition of RPL17 expression suppressed CRC cell growth and long-term colony formation by inducing apoptotic cell death. Similarly, targeting RPL17 effectively suppressed tumor formation in a mouse xenograft model. RNA sequencing of RPL17-silenced CRC cells revealed the same directional regulation of 159 (93 down- and 66 up-regulated) genes. Notably, NIMA-related kinase 2 (NEK2), which functionally cooperates with extracellular-regulated protein kinase (ERK) and plays a pivotal role in mitotic progression and stemness maintenance, was down-regulated. RPL17 silencing reduced NEK2, β-catenin, and p-ERK protein levels. These molecular alterations reflected the reduction in sphere-forming capacity, expression of stem cell marker genes, migration, and invasion. Reversely, RPL17 overexpression increased the ability of long-term colony formation, migration, and invasion.

**Conclusion:** Our findings indicate that RPL17 promotes CRC proliferation and stemness via the ERK and NEK2/β-catenin signaling axis, and targeting RPL17 could be the next molecular strategy for both primary CRC treatment and prevention of secondary tumor formation.

## Introduction

Colorectal cancer (CRC) is the third most frequent cancer globally and the second major cause of cancer-associated death 1. Patients diagnosed with CRC commonly develop colorectal metastases, and about 80% to 90% of these patients are diagnosed with unresectable metastatic liver disease 2. A critical challenge of CRC is metastasis and recurrence; systemic treatment of metastatic CRC with targeted agents is a rapidly evolving field 3.

Human ribosomes consist of four different rRNAs and 80 ribosomal proteins (RPs). RPs are in conjunction with rRNA structures for cellular translation processes and to promote accurate folding of rRNA during ribosome assembly 4. However, recent evidences indicate that apart from involvement in protein synthesis, RPs perform extra-ribosomal functions, which include transcriptional regulation, DNA repair, RNA splicing, cell proliferation, regulation of apoptosis, and development 5-7. For example, inhibition of many RPs leads to p53 accumulation, cell death, and defective development 7.

In terms of tumor biology, various RPs are known to be overexpressed in several cancer types and are closely related to cancer progression and metastasis. For instance, ribosomal protein S13 (RPS13) promotes cell cycle progression and cell growth by inhibiting p27 in gastric cancer 8. Phosphorylated RPS3 and anti-apoptotic protein TRAF2 enhance radiation resistance in non-small cell lung cancer (NSCLC) 9. In addition, puromycin induces apoptosis by promoting binding of RPL5 and RPL11 to MDM2 which in turn restores p53 activity in CRC and NSCLC cells 10. Moreover, we have previously evidenced that ribosomal protein L9 regulates CRC growth via the Id-1/NF-κB signaling axis 11.

Ribosomal protein L17 (RPL17) is a 60S subunit component that is affiliated with the L6P family of RPs. We found its expression to be increased in a series of solid colorectal tumors as well using the GEO public database (accession number GSE10972) 12. Despite its altered expression in CRC tissues, the functional role of RPL17 in colorectal tumor progression has not been defined yet.

Meanwhile, it is well known that a cancer stem cell (CSC) subset is responsible for tumor initiation, migration, recurrence, chemo-resistance, and radio-resistance. We have also previously reported that the up-regulation of the CSC marker CD133 by STAT3 transcription factor promotes liver tumor development 13. Identification and characterization of functional pathways or biomarkers associated with CSC biology will provide useful information for developing novel treatment strategies against both tumor invasion and recurrence.

The purpose of this study was to explore and identify the novel roles of RPL17 in CRC progression. To address this, we first observed whether inhibition of RPL17 expression using target-specific siRNA could change CRC phenotypes *in vitro* and *in vivo*. We also examined the molecular mechanisms underlying the therapeutic response induced by RPL17 silencing. Here, we found that RPL17 promotes CRC proliferation and stemness via the ERK and NEK2/β-catenin signaling axis, and targeting RPL17 could be the next molecular strategy for CRC treatment.

## Materials and methods

### Cell culture and siRNA transfection

Human CRC cell lines, HCT116, HT29, SW480 and DLD-1, were purchased from the Korean Cell Line Bank (KCLB, Seoul, Republic of Korea) and cultured in RPMI-1640 medium supplemented with 10% fetal bovine serum (FBS) and 1% penicillin/streptomycin solution (all from Welgene, Daegu, Republic of Korea). Prior to siRNA transfection, cells were plated at 30% density for 24 h. siRNAs (15 nM) and Lipofectamine 2000 (Invitrogen, Carlsbad, CA, USA) were mixed in Opti-MEM (Thermo Scientific, Rockford, IL, USA) and added to cells for 5 h. RPL17-specific siRNA and negative control siRNA (NCsiRNA) duplexes were purchased from Ambion (ID# s12179, Austin, TX, USA) and Bioneer (Daejeon, South Korea), respectively. The sequences of NCsiRNA were as follows: 5ʹ- ACGUGACACGUUCGGA GAA(UU)-3ʹ (sense) and 5ʹ-UUCUCCGAACGUGUCACGU-3ʹ (antisense).

### Cell proliferation and clonogenic assay

The cell viability assay was performed using the Cell Counting Kit-8 (Dojindo, Kumamoto, Japan) according to the manufacturer's instructions. For the clonogenic assay, siRNA-transfected cells and RPL17-overexpressing stable cells were seeded into three independent wells of 6-well culture plates (1×10^3^ and 5×10^2^ cells per well, respectively) and incubated in medium for 12 days. After fixation in methanol, viable colonies were stained with 0.5% crystal violet for 30 min and counted under a microscope.

### RNA isolation and quantitative real-time PCR (qRT-PCR)

Alterations in gene expression at the mRNA level were analyzed using qRT-PCR. Total RNA was isolated using TRIzol (Ambion) and synthesized into cDNA using the 1st Strand cDNA Synthesis Kit (Takara Biotech, Kusatsu, Shiga, Japan) as per the manufacturer's instructions. cDNAs of RPL17 knockdown genes were amplified using corresponding pair of primers (RPL17 forward, 5'-GTGTACCATTCCGACGTTACAA-3' and RPL17 reverse, 5'-TCTGCGTTTTTAAGCATGTGC-3'; GAPDH forward, 5'-GGGAGCCAAAAGGGTCATCATCTC-3' and GAPDH reverse, 5'-CCATGCCAGTGAGCTTCCCGTTC-3') synthesized by Macrogen (Seoul, Republic of Korea). Relative mRNA expression was evaluated and quantified using the LightCycler 96 system (Roche, Basel, Switzerland) according to the instruction manual from the manufacturer. GAPDH mRNA levels were used for normalization.

### Cell cycle analysis and detection of apoptosis

Cells were transfected with siRNA as described in section 2.1, but were cultured in 60-mm petri dishes and harvested 48 h after siRNA transfection. To analyze the percentage of cells in each cell cycle phase, the cells were washed with cold phosphate-buffered saline (PBS) and then fixed for 24 h with 70% cold ethanol at -20 ℃. Cells were washed again and cultured in the dark with propidium iodide (PI) solution containing RNase A (BD Biosciences, San Diego, CA, USA) for 30 min at room temperature. For the detection of apoptosis, the cells were washed with cold PBS and stained with both Annexin V and PI using the FITC Annexin V Apoptosis Detection Kit I (BD Bioscience) according to the manufacturer's instructions. Cell death and cell cycle analyses were conducted using the FACS Canto II flow cytometer (BD Biosciences) and quantified using the FACSDiva software program.

### Generation of RPL17-overexpressing CRC cell line

To establish a cell line overexpressing RPL17, SW480 cells were transfected with pCMV6-entry vector (vector only) or RPL17-expressing vector (v-RPL17) (all from OriGene, Rockville, MD, USA) using Lipofectamine 2000 (Invitrogen). Cells were selected for resistance to 800 µg/mL of Geneticin (Thermo Fisher Scientific, Waltham, MA, USA), and the geneticin-resistant clones were obtained by single clonal selection. We selected #13 clone for all subsequent experiments.

### Tumorigenic assay in nude mice

HCT116 cells were transfected with NCsiRNA or RPL17siRNA using Lipofectamine 2000 (Invitrogen). After 24 h of treatment, the silenced cells were harvested and mixed with Matrigel (Corning, NY, USA). A total of 1 × 10^6^ cells were injected subcutaneously into the left and right flanks of four-week-old male BALB/c nude mice (Orientbio, Seongnam, Korea). Tumor size was measured for 31 days at 2-3 day intervals using a Vernier caliper (n=9 per group). At the end of observation, the tumor weight of each mouse was evaluated.

### RNA sequencing

RNA sequencing was performed as described previously 11. The datasets generated and analyzed in this study are accessible in the GEO database with the accession number GSE78195.

### Western blotting

Equal amounts of total protein were fractionated by sodium dodecyl sulfate polyacrylamide gel electrophoresis on a 10% gel and transferred to a polyvinylidene fluoride membrane (Roche). The membranes were blocked with 5% milk/Tris-buffered saline plus Tween 20 (TBST) and incubated with primary antibodies against PARP-1 (sc-8007), NEK2 (sc-55601), β-actin (sc-47778) (all from Santa Cruz Biotechnology, Santa Cruz, CA, USA), pro-caspase-3 (#9662), β-catenin (#9562), phospho-ERK (#9101), ERK (#9102) (all from Cell Signaling, Danvers, MA, USA), and RPL17 (ab155781, Abcam, Cambridge, UK). HRP goat anti-mouse IgG, HRP goat anti-rabbit IgG, and HRP rabbit anti-goat IgG (Santa Cruz) were used as secondary antibodies. Immunoreactive bands were visualized using the LAS-3000 Imager (Fujifilm Corporation, Tokyo, Japan).

### Sphere-forming assay

HT29 cells (1 × 10^4^) were suspended in serum-free DMEM/F-12 medium containing 4 ng/mL insulin (Invitrogen), 2% B27 (Invitrogen), 20 ng/mL epidermal growth factor, and 10 ng/mL basic fibroblast growth factor, and then seeded into 24-well ultralow attachment plates (Corning). After plating the single cells, cells were transfected with NCsiRNA or RPL17siRNA using Lipofectamine RNAiMAX (Invitrogen) according to the manufacturer's protocol. The number of cell spheres was counted on day 7 of siRNA transfection.

### Invasion and migration assay

Invasion assay was performed using Corning Matrigel invasion chamber 24-well plate (#354480, Corning). NCsiRNA- or RPL17siRNA-transfected HCT116 cells (1 × 10^5^) were suspended in 200 μL medium and plated in the upper chamber. The lower chamber was filled with 600 μL of medium containing FBS as an attractant. After 24 h, the cells were fixed and stained with 0.5% crystal violet. Images were collected at ×100 magnification under a phase-contrast microscope. The migration assay was performed in the same way as the invasion assay, except using Transwell (#3422, Corning).

### Statistical analysis

**Figure 1:**
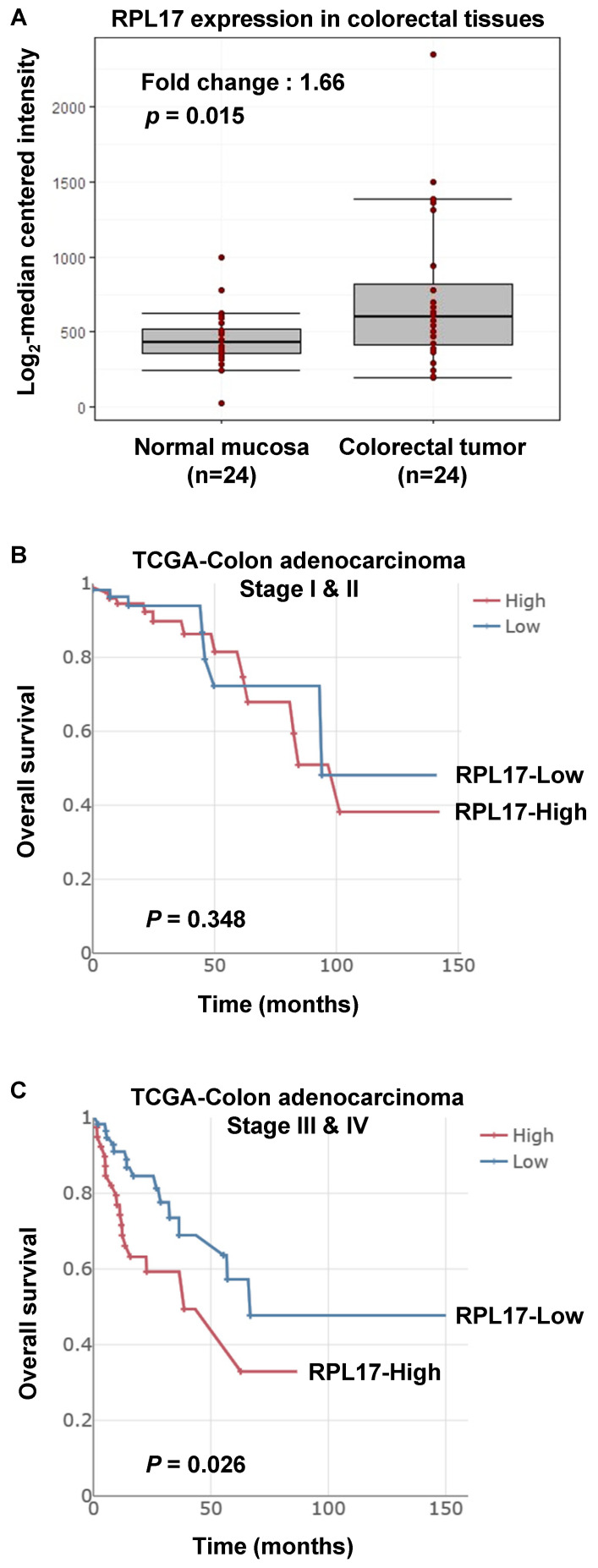
Increased expression of RPL17 and its correlation to prognosis in CRC patients. (A) Relative RPL17 expression in matched normal mucosa (*n* = 24) vs colorectal tumor tissues (*n* = 24). The data were extracted and modified from GEO public database (accession number GSE10972). Fold change: 1.66, *P* value: 0.01586 by two-sample *t*-test. (B,C) Associations between RPL17 expression and overall survival in the stage I & II patient's group (B) or in the stage III & IV patient's group (C).

All statistical analyses were performed using the Student's *t*-test, except for RPL17 expression comparative data between normal and tumor tissues (Fig. 1), which was performed using the two-sample *t*-test. All results are presented as the mean ± standard error of mean (SEM). P-values < 0.05 (*) and < 0.01 (**) were considered statistically significant.

## Results

### Big data analysis of RPL17 expression and its prognostic value in CRC

From the data investigating global gene expression in 24 human colorectal tumors against matched normal mucosa 12, we found that the average expression of RPL17 is upregulated in tumor tissues (Fig. 1A). To further verify the biological importance of dysregulation of RPL17 in mediating CRC initiation and/or progression, we have also analyzed out the relationship between RPL17 overexpression and its prognostic value from TCGA-Colon adenocarcinoma big data. As shown in Figure 1B and 1C, higher expression of RPL17 mRNA exhibited a poorer overall survival in patient group of Stage III & IV than of Stage I & II, indicating that biological impact of RPL17 overexpression is gradually increased as the disease progresses.

### RPL17 knockdown inhibits proliferation and colony formation of CRC cells

**Figure 2:**
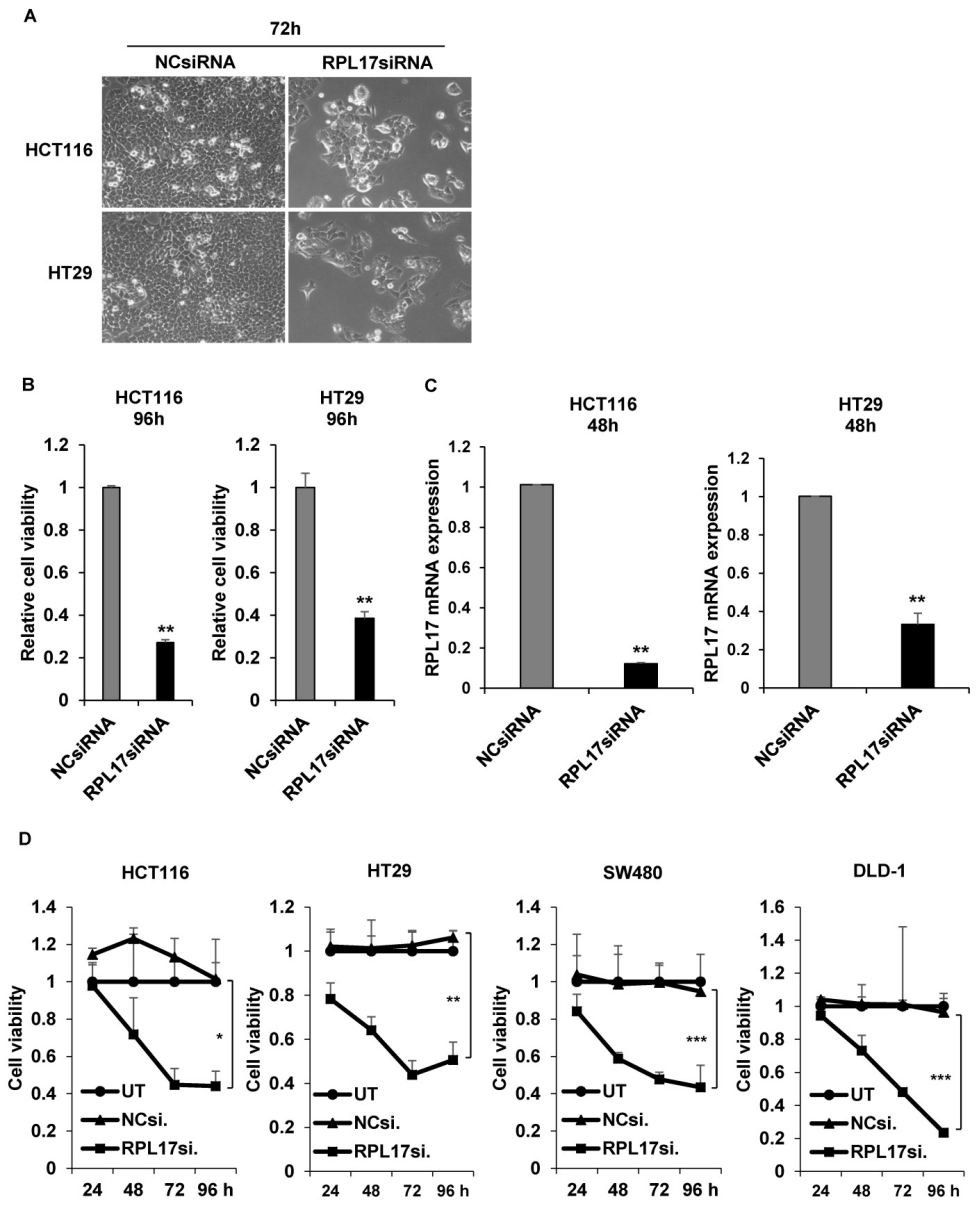
siRNA knockdown of RPL17 inhibits the growth of CRC cells. (A) Representative light microscopy images of HCT116 and HT29 cells 72 h after transfection. (B) Detection of relative cell viability of HCT116 and HT29 cells 96 h after siRNA transfection. (C) Detection of RPL17 mRNA expression in HCT116 and HT29 cells 48 h after transfection. (D) Daily measurement of cell viability of HCT116, HT29, SW480 and DLD-1 cells after siRNA transfection. UT, untreatment; NCsiRNA, negative control siRNA; RPL17siRNA, RPL17-specific siRNA. *p < 0.05; **p < 0.01 vs control.

**Figure 3:**
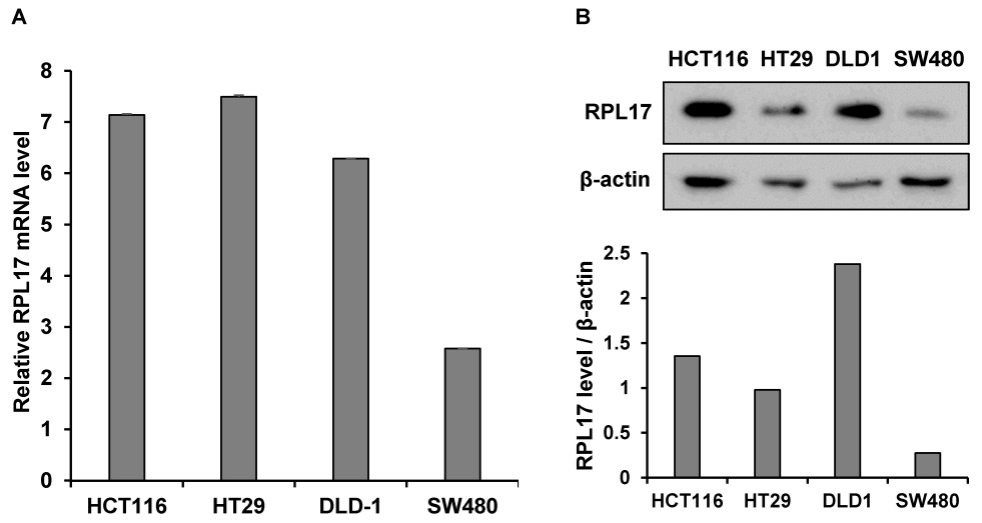
Relative basal expression of RPL17 in diverse CRC cell lines at mRNA (A) or protein (B) level. β-actin protein was included as a loading control.

**Figure 4:**
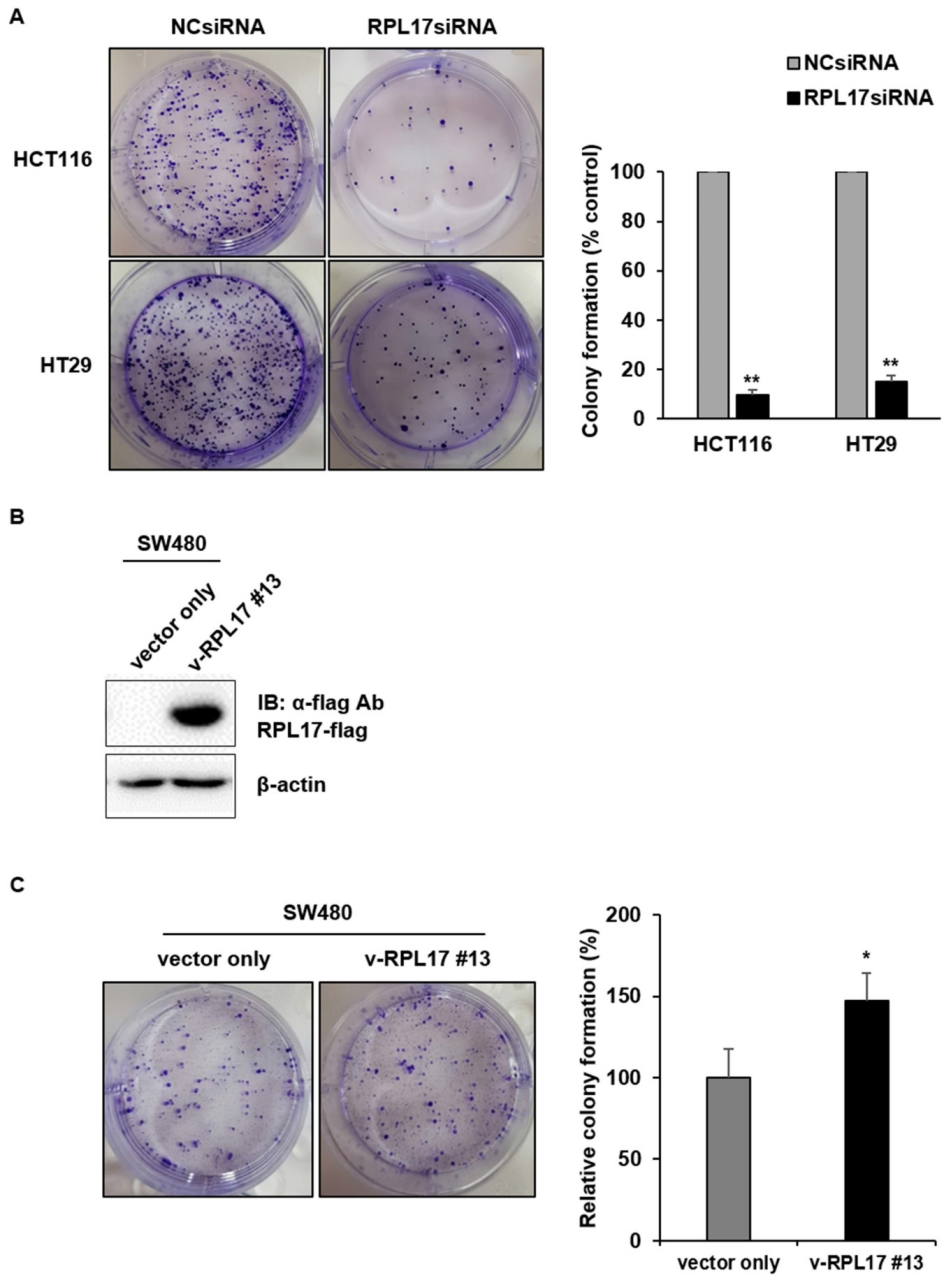
RPL17 promotes long-term colony formation of CRC cells. (A) Observation of long-term colony formation of HCT116 and HT29 cells 10 days after transfection. The number of colonies was counted and graphed for an effective comparison. The data represent three independent experiments. (B) Detection of the RPL17-overexpressing SW480 cell line (v-RPL17 #13). β-actin was used as a loading control. (C) Observation of long-term colony formation of SW480-control cells (vector only) and v-RPL17 #13 cell line. *p < 0.05; **p < 0.01 vs control.

To detect the effect of RPL17 knockdown about CRC cell growth, we transfected HCT116 and HT29 cells using a NCsiRNA or RPL17siRNA. After 72 h, we observed using a microscope that the cell viability of RPL17 knockdown cells was significantly reduced (Fig. 2A). Relative cell viability was also assessed using an MTT assay. The proliferation of RPL17siRNA-treated cells was reduced by approximately 70% and 60% in HCT116 and HT29 cells, respectively, when compared with those of the control group (Fig. 2B). Consistent with the changes in cell growth, RPL17siRNA effectively silenced the target gene expression in both HCT116 and HT29 cells (Fig. 2C). Significantly, the growth suppressing effect on HCT116 and HT29 cells was reproduced in two additional CRC cells SW480 and DLD-1 to a varying degree (Fig. 2D). Meanwhile, to validate the biological function of RPL17 in CRC growth, a CRC cell line overexpressing RPL17 was generated. We first detected the basal level of RPL17 expression in four CRC cell lines and observed that SW480 cells expressed RPL17 at the lowest level in both RNA and protein levels (Fig. 3A and 3B). Thus, we utilized SW480 cells for all the subsequent overexpression experiments. Concordance with the short-term silencing results, at day 10, RPL17 knockdown inhibited the long-term colony formation of HCT116 and HT29 cells by about above 90% and 80%, respectively (Fig. 4A). Conversely, SW480 clones overexpressing RPL17 (Fig. 4B) had increased colony forming ability when compared to control vector transfected cells (Fig. 4C). These results indicate that RPL17 is functionally involved to CRC cell survival.

### Targeting RPL17 impairs cell cycle progression and induces apoptosis

**Figure 5:**
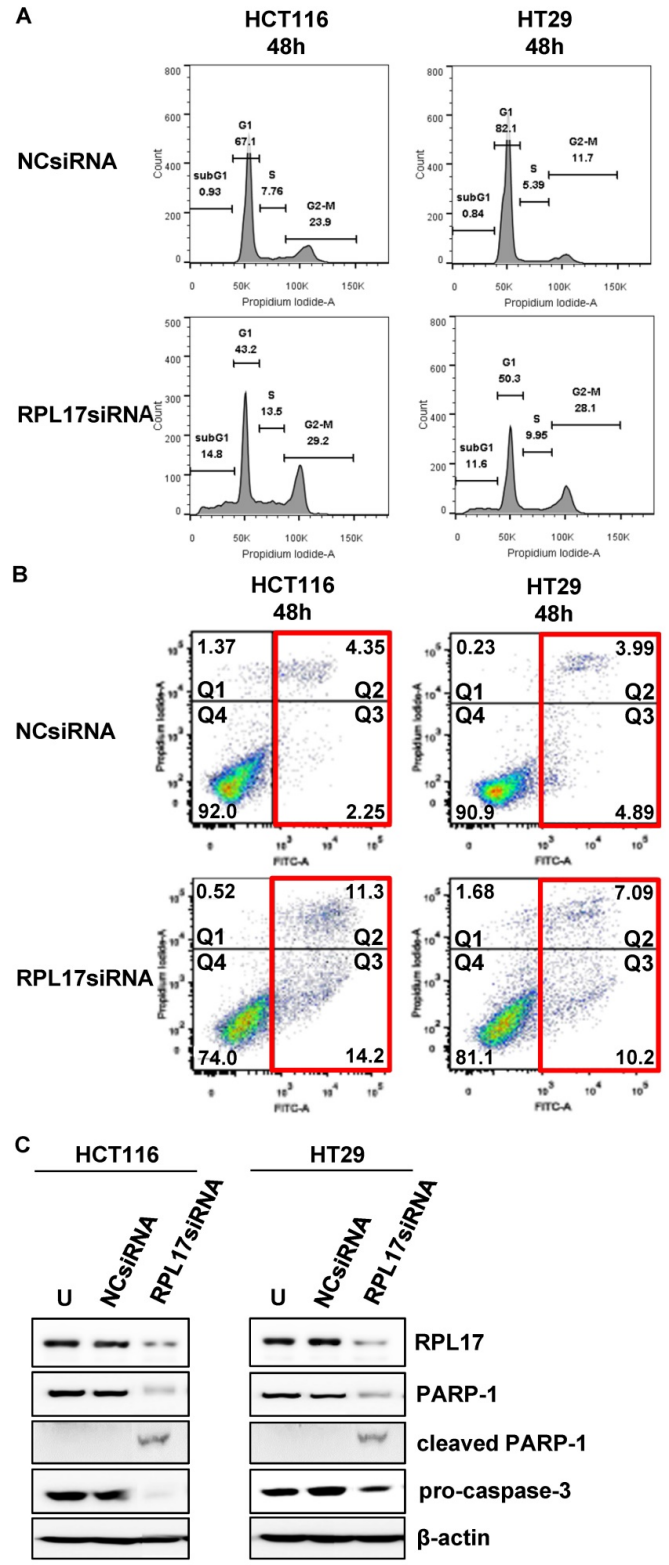
Targeting RPL17 increases the sub-G1 population and apoptosis of CRC cells. (A) Changes in cell cycle progression were measured by flow cytometry 48 h after siRNA treatment and is shown in a histogram. (B) The fraction of apoptotic cells was measured by FACS analysis 48 h after siRNA treatment. (C) Protein expression of RPL17, PARP-1, cleaved PARP-1, and pro-caspase 3 in HCT116 and HT29 cells. β-actin was used as a loading control. U, untreated cells.

We then confirmed whether the inhibition of CRC cell growth by RPL17 knockdown was due to delay of cell cycle progression and/or induction of apoptotic cell death. In comparison with the control group, silencing of RPL17 increased the percentage of sub-G1 phase cells in both HCT116 and HT29 cells (Fig. 5A). Because the sub-G1 population contains apoptotic cells, the phenomenon was then validated by staining HCC cells with Annexin V, an apoptotic marker. This showed that silencing of RPL17 increased the number of apoptotic cells at 48 h after transfection (Fig. 5B). The percentages of late (Q2 portion) plus early apoptotic cell populations (Q3 portion) HCT116 and HT29 cells were increased by about 3.8-fold and 1.9-fold, respectively. In a molecular level, this condition induced an accompanying reduction in the protein level of PARP-1 and pro-caspase 3 (Fig. 5C). These results show that the inhibition of CRC cell proliferation by RPL17 silencing was incurred by block of cell cycle progression and induction of apoptosis.

### Silencing of RPL17 expression suppresses colorectal tumor growth in vivo

**Figure 6:**
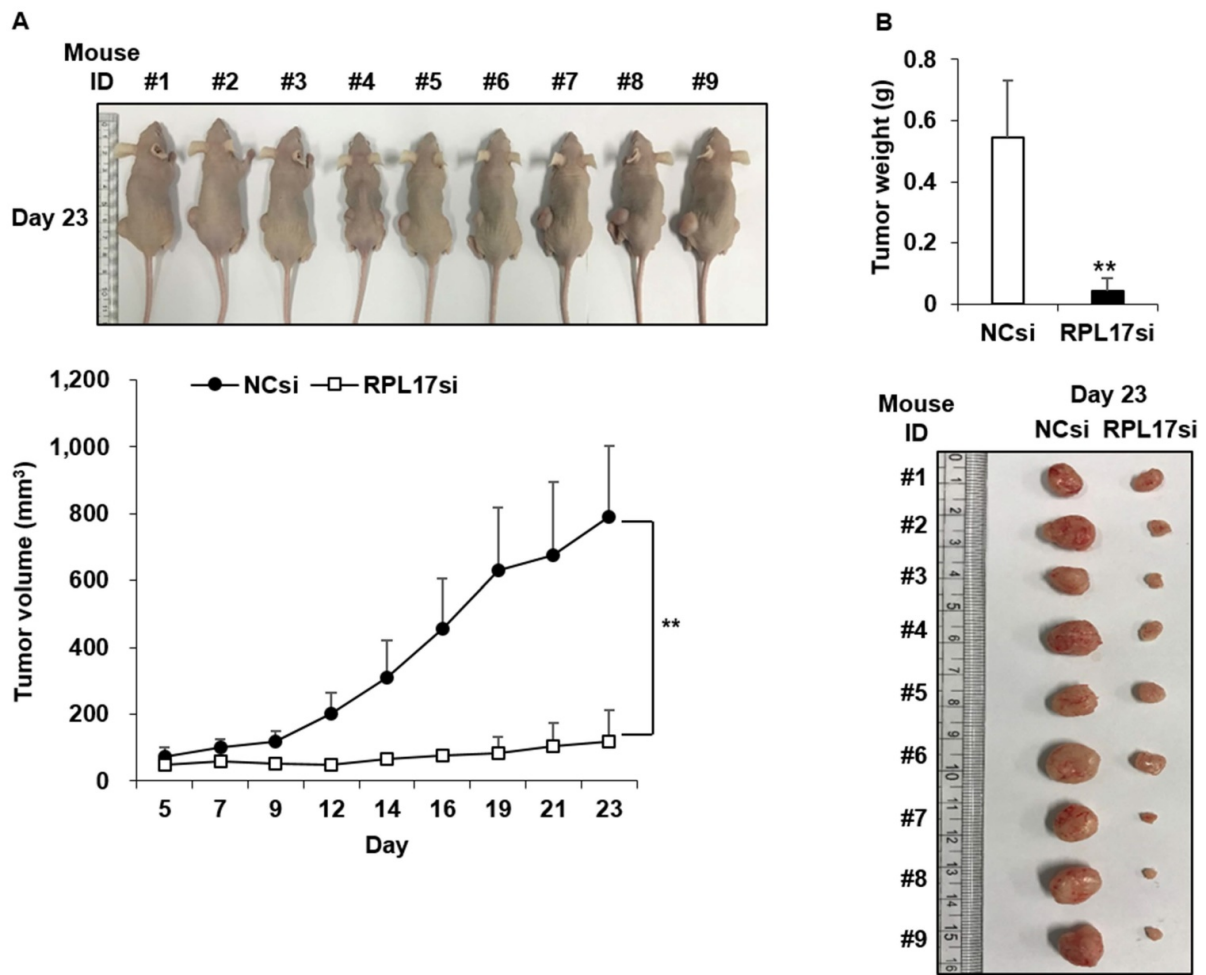
Antitumor effect of RPL17siRNA on subcutaneous growth of HCT116 xenografts. (A) Kinetics of tumor growth. (B) Gross tumor morphology and average tumor weight in each siRNA treatment group 23 days after inoculation of tumor cells. **p < 0.01 vs control.

To affirm the suppressive effect of RPL17 silencing in vivo, a tumor formation assay was performed in BALB/c nude mice. HCT116 cells treated with NCsiRNA or RPL17siRNA were subcutaneously inoculated into the left and right flank, respectively, and then the size of each tumor was measured for 23 days at 2-3 day intervals. Nine days after the inoculation, there was a clear difference in tumor sizes between the control and RPL17 knockdown, such that the tumor sizes of the control were larger (Fig. 6A). On day 23, a significant difference was evident in the tumor size and weight of the control and RPL17 knockdown group (Fig. 6B).

### RPL17 is functionally associated with ERK and NEK2/β-catenin signaling axis

**Figure 7:**
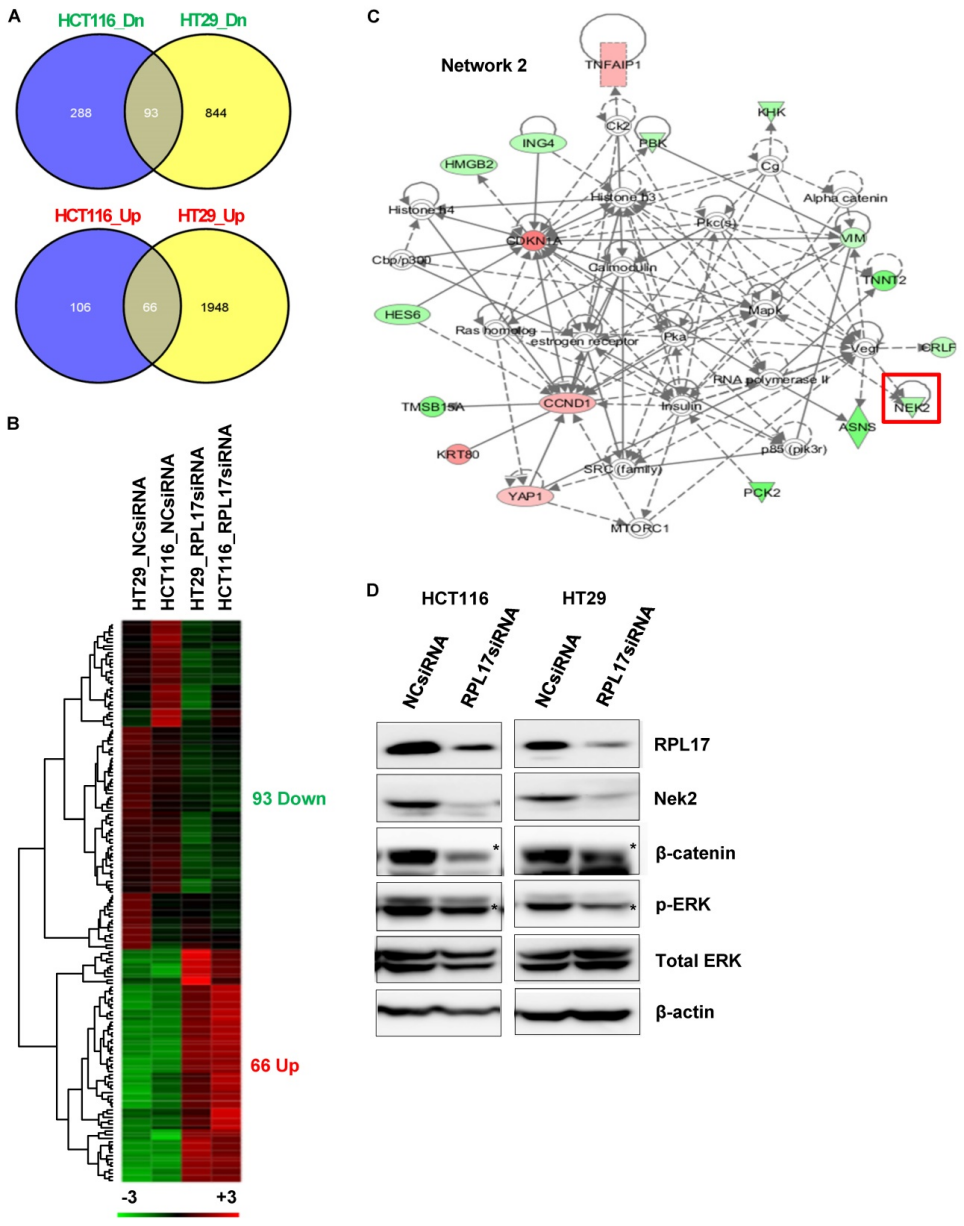
Molecular mechanisms underlying CRC phenotypic changes induced by RPL17 silencing. (A)-(C) Transcriptomic analysis of RPL17 knockdown cells by RNA sequencing. (A) The number of genes dysregulated in HCT116 and/or HT29 cells 48 h after siRNA treatment. (B) A heat map of the 159 genes commonly up- or down-regulated in both HCT116 and HT29 cells (Red, up-regulated; green, down-regulated). (C) IPA network 2 functionally associated with NEK2. (D) Detection of RPL17, NEK2, β-catenin, phospho-ERK, and total ERK in HCT116 and HT29 cells 48 h after siRNA transfection.

To find out the molecular mechanisms by which RPL17 silencing could induce the observed phenotypic changes, we performed RNA sequencing to compare the patterns of global gene expression in RPL17-silenced HCT116 and HT29 cells to those of NCsiRNA-treated control cells. When defined at least a 2-fold change, silencing of RPL17 disturbed the expression of 553 RNA transcripts (172 up-regulated and 381 down-regulated) in HCT116 cells and 2,951 RNA transcripts (2,014 up-regulated and 937 down-regulated) in HT29 cells, respectively (Fig. 7A). Overlapping of these two gene sets generated a commonly dysregulated list of 159 (93 down- and 66 up-regulated) genes (Fig. 7B). Ingenuity pathway analysis (IPA) showed that the 159 genes were functionally enriched in the top five networks (Supplementary Fig. 1). Notably, as shown in Fig. 7C, we found that the expression level of NEK2, which is involved in mitotic regulation, drug resistance and cancer stemness 14-17, was down-regulated. Consistent with our observation, it was previously demonstrated that NEK2 silencing decreases proliferation and induces apoptosis in CRC cells 18 and that NEK2 regulates Wnt/β-catenin signaling pathway which plays a pivotal role in exerting cancer stemness 14, 19. It was also found that ERK is functionally associated with NEK2 expression and knockdown of NEK2 inhibits ERK phosphorylation in gastric cancer cells 20. Western blotting validated that silencing of RPL17 expression reduces the levels of NEK2/β-catenin and the activity of ERK in both HCT116 and HT29 cells (Fig. 7D). These findings indicate that the function of RPL17 is associated with Nek2/β-catenin and ERK signaling in CRC cells.

### Targeting RPL17 reduces CRC stemness

**Figure 8:**
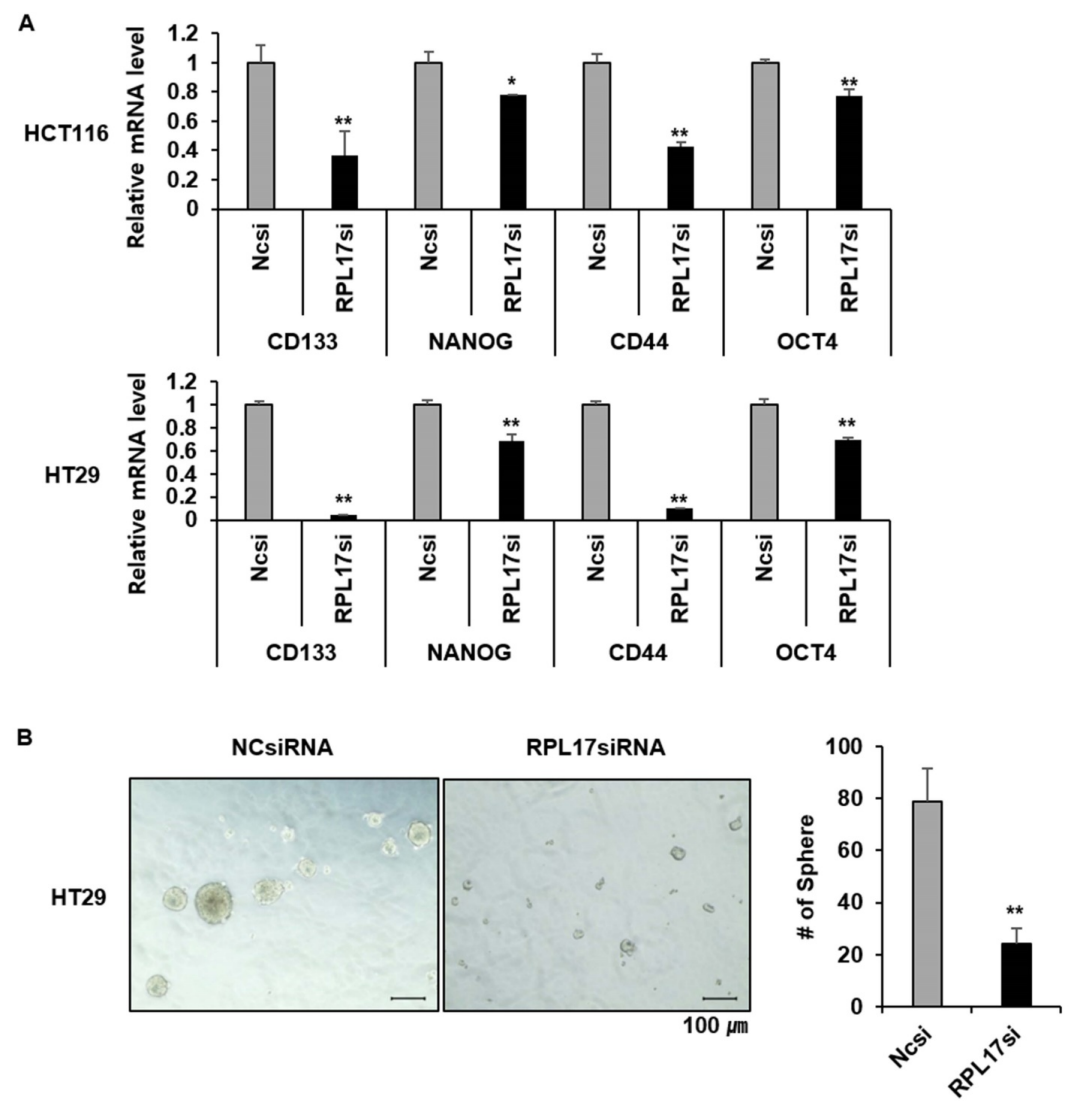
Targeting RPL17 reduces the expression of cancer stemness genes and sphere-forming capacity. (A) mRNA levels of CD133, NANOG, CD44, and OCT4 in CRC cells treated with NCsiRNA or RPL17siRNA. It is expressed as fold changes relative to NCsiRNA. (B) Representative microscopy images of sphere formation of NCsiRNA- or RPL17siRNA-transfected HT29 cells. The number of spheres were counted and graphed for an effective comparison. *p < 0.05; **p < 0.01 vs control.

**Figure 9:**
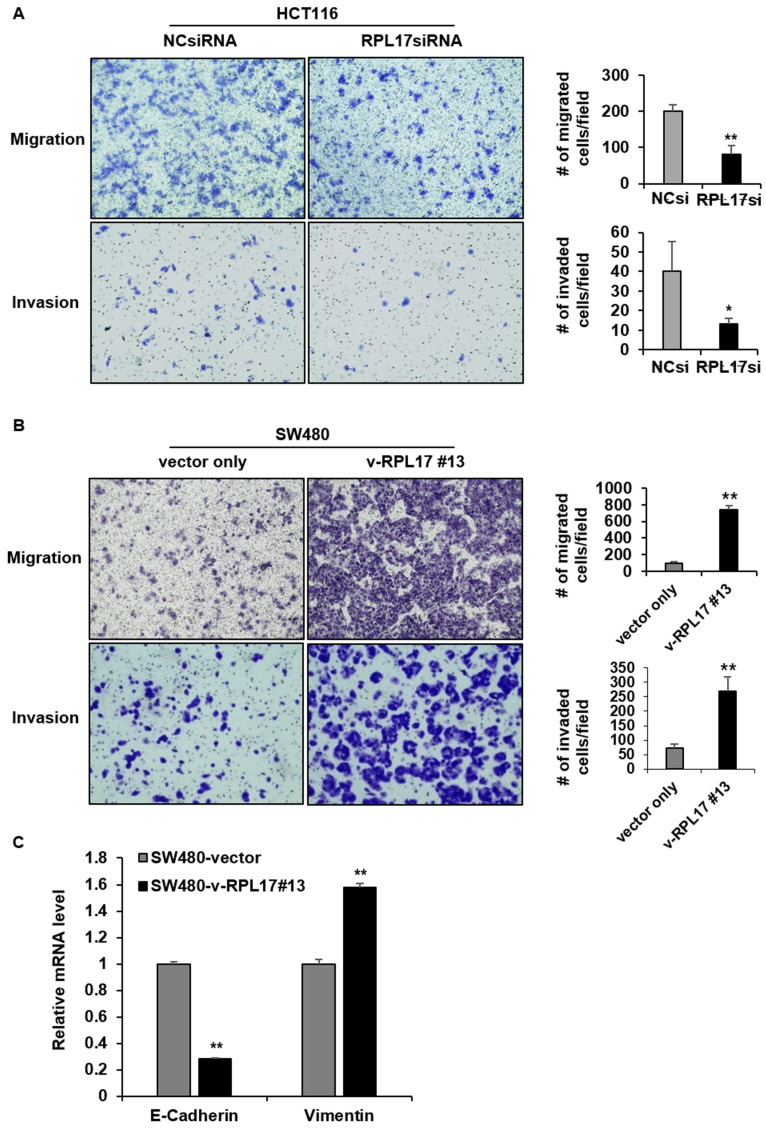
RPL17 promotes migration and invasion of CRC cells. (A) Representative light microscopy images of migratory or invasive NCsiRNA- or RPL17siRNA-transfected HCT116 cells. The number of migrated or invaded cells was counted and graphed. (B) Representative light microscopy images of migratory or invasive control (vector only) or v-RPL17 #13 cells. (C) Changes in mRNA level of E-cadherin and vimentin in control or v-RPL17 #13 cells. *p < 0.05; **p < 0.01 vs control.

Given the functional significance of Nek2/β-catenin signaling in CSC biology and the observation of Nek2/β-catenin repression under RPL17 knockdown condition, we have determined to investigate if targeting of RPL17 could reduce colorectal CSC properties by detecting the changes in expression of cancer stemness marker genes, sphere forming capacity, migration ability and invasion ability. As expected, RPL17 knockdown resulted in reduction of expressions of the representative stemness markers such as CD133, NANOG, CD44 and OCT4 in both HCT116 and HT29 cells (Fig. 8A). These molecular alterations reflected to reduction of sphere forming capacity in the CSC subset of HT29 parental cell population (Fig. 8B). Furthermore, RPL17 knockdown reduced the numbers of migrated and invaded cells as well in HCT116 population (Fig. 9A). Reversely, overexpression of RPL17 increased the abilities of migration and invasion in SW480 cells (Fig. 9B), accompanying with downregulation of E-cadherin and upregulation of vimentin (Fig. 9C). These data implicate a possibility that targeting RPL17 could be utilized as a novel option to therapeutically eliminate stemness in the CRC microenvironment.

## Discussion

RPs are components of ribosomes and are crucial in protein biosynthesis. However, some of these RPs are upregulated in many types of human tumor tissues compared to surrounding normal tissues, and are associated with tumorigenesis, cell migration and invasion, and genomic integrity 7, 21. Over 30 RPs are known to exert extra-ribosomal functions, and more than 10 RPs have shown to regulate cell growth and apoptosis in CRC cells 5, 7, 22, 23. Despite the enhanced RPL17 expression in colorectal tumor progression (Fig. 1), the fundamental mechanisms underlying the deregulation of RPL17 remain to be defined. Here, we evidenced that RPL17 knockdown suppresses CRC cell proliferation and long-term colony formation through a strong induction of apoptosis, and that overexpression of RPL17 converses the effect (Figs. 2-5), which demonstrates that RPL17 is important for the growth of CRC. Additionally, a mouse xenograft model showed that targeting RPL17 significantly inhibited tumor growth (Fig. 6).

We next sought to find a molecular explanation of how RPL17 functions in tumor progression. The phenotypic changes triggered by RPL17 silencing were associated with a common and coordinated dysregulation of 159 genes (Fig. 7B). These data indicate that although RPL17 is involved in protein synthesis, it does not affect entire cellular protein biosynthesis, it is rather gene-specific and protein-specific. Of all the genes that were dysregulated by RPL17 knockdown, oncogenic NEK2, which is known to regulate ERK phosphorylation for cell survival 20, was down-regulated in network 2 of the IPA (Fig. 7C). Recent evidences support the novel notion that CRC initiation can be driven by a CSC subset, which is responsible for tumor persistence, relapse, metastasis, chemo-resistance, and radio-resistance, thereby establishing CSCs as important therapeutic targets 24. There is increasing appreciation that NEK2 plays a pivotal role in cancer cell proliferation and stemness. For example, overexpression of NEK2 correlates with poor survival in CRC 25, 26 and NEK2 regulates stem-like properties in HCC 16. We confirmed by western blotting that the protein level of NEK2 was lower in RPL17 knockdown HCT116 and HT29 cells than the control (Fig. 7D). In addition, NEK2 promotes mitosis and cancer stemness by activating Wnt/β-catenin signaling 14, 16. Hence, we investigated the change in β-catenin activity in RPL17 knockdown HCT116 and HT29 cells and observed that the protein level of β-catenin was also decreased. Moreover, RPL17 knockdown cells exhibited decreased abilities in sphere-formation, migration, and invasion, and RPL17 overexpression reversed these phenomena (Figs. 8 and 9). These results indicate that RPL17 targeting causes inactivation of NEK2 and β-catenin, which leads to the inhibition of stemness in CRC cells. This mechanism, whereby the RPL17 inhibitor interferes with NEK2/β-catenin signaling, may be of significance for the clinical use of RPL17 inhibitors and requires thorough investigation.

**Figure 10:**
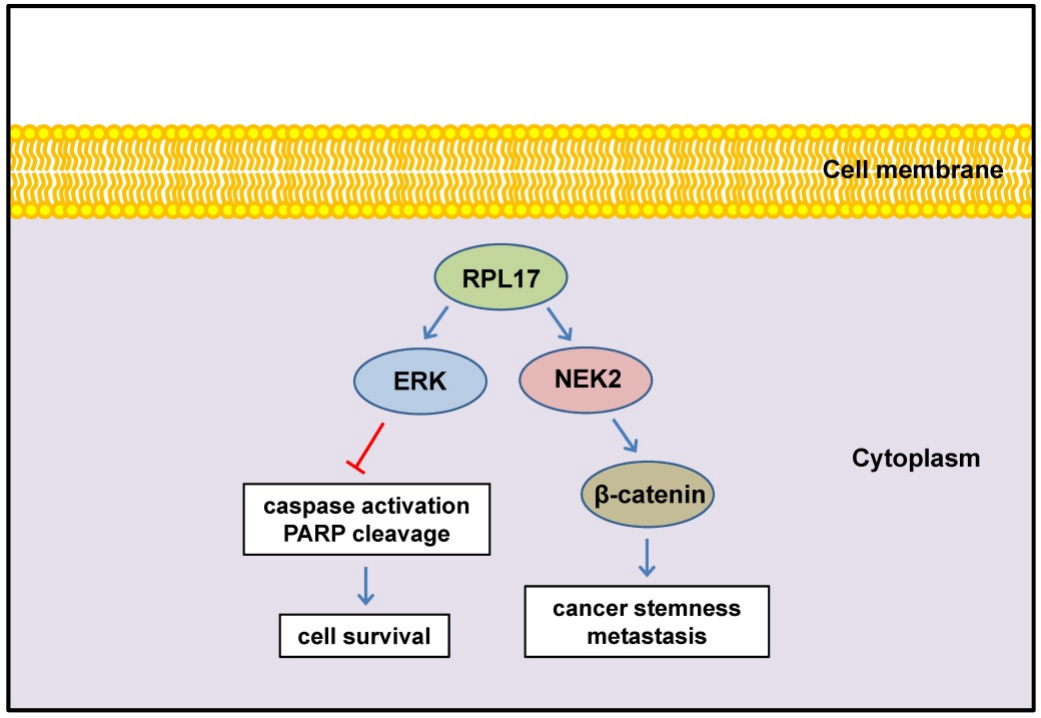
Schematic diagram illustrating the regulation of RPL17 to exert cancer cell survival and stemness.

In conclusion, we defined here for the first time that enhanced expression of RPL17 cooperates with NEK2/β-catenin and ERK signaling to promote CRC progression and to augment stemness (Fig. 10). Thus, RPL17 targeting could be the next molecular strategy for both primary CRC treatment and prevention of metastasis or recurrence in the colorectal tumor microenvironment.

## Supplementary Material

Supplementary figure.

## References

[1] Bray F, Ferlay J, Soerjomataram I (2018). Global cancer statistics 2018: GLOBOCAN estimates of incidence and mortality worldwide for 36 cancers in 185 countries. CA Cancer J Clin.

[2] Benson AB 3rd, Venook AP, Bekaii-Saab T (2014). Colon Cancer, Version 3. J Natl Compr Canc Netw.

[3] Seeber A, Gastl G (2016). Targeted therapy of colorectal cancer. Oncol Res Treat.

[4] Taylor DJ, Devkota B, Huang AD (2009). Comprehensive molecular structure of the eukaryotic ribosome. Structure.

[5] Warner JR, McIntosh KB (2009). How common are extra-ribosomal functions of ribosomal proteins?. Mol Cell.

[6] Lai MD, Xu J (2007). Ribosomal proteins and colorectal cancer. Curr Genomics.

[7] Heras-Rubio ADL, Perucho L, Paciucci R (2014). Ribosomal proteins as novel players in tumorigenesis. *Cancer Metastasis Rev*.

[8] Guo X, Shi Y, Gou Y (2011). Human ribosomal protein S13 promotes gastric cancer growth through down-regulating p27Kip1. J Cell Mol Med.

[9] Yang HJ, Youn H, Seong KM (2013). Phosphorylation of ribosomal protein S3 and antiapoptotic TRAF2 protein mediates radioresistance in non-small cell lung cancer cells. J Biol Chem.

[10] Jung JH, Lee H, Kim JH (2019). p53-Dependent apoptotic effect of puromycin via binding of ribosomal protein L5 and L11 to MDM2 and its combination effect with RITA or doxorubicin. *Cancers*.

[11] Baik IH, Jo GH, Seo D (2016). Knockdown of RPL9 expression inhibits colorectal carcinoma growth via the inactivation of Id-1/NF-κB signaling axis. Int J Oncol.

[12] Jiang X, Tan J, Li J (2008). DACT3 is an epigenetic regulator of Wnt/beta-catenin signaling in colorectal cancer and is a therapeutic target of histone modifications. Cancer Cell.

[13] Won C, Kim BH, Yi EH (2015). Signal transducer and activator of transcription 3-mediated CD133 up-regulation contributes to promotion of hepatocellular carcinoma. Hepatology.

[14] Mbom BC, Siemers KA, Ostrowski MA (2014). Nek2 phosphorylates and stabilizes β-catenin at mitotic centrosomes downstream of Plk1. Mol Biol Cell.

[15] Zhou W, Yang Y, Xia J (2013). NEK2 induces drug resistance mainly through activation of efflux drug pumps and is associated with poor prognosis in myeloma and other cancers. Cancer Cell.

[16] Lin S, Zhou S, Jiang S (2016). Nek2 regulates stem-like properties and predicts poor prognosis in hepatocellular carcinoma. Oncol Rep.

[17] Fang Y, Zhang X (2016). Targeting NEK2 as a promising therapeutic approach for cancer treatment. Cell Cycle.

[18] Suzuki K, Kokuryo T, Senga T (2010). Novel combination treatment for colorectal cancer using Nek2 siRNA and cisplatin. Cancer Science.

[19] Fu SJ, Chen J, Ji F (2017). MiR-486-5p negatively regulates oncogenic NEK2 in hepatocellular carcinoma. Oncotarget.

[20] Fan WD, Chen T, Liu PJ (2019). NIMA related kinase 2 promotes gastric cancer cell proliferation via ERK/MAPK signaling. World J Gastroenterol.

[21] Xu X, Xiong X, Sun Y (2016). The role of ribosomal proteins in the regulation of cell proliferation, tumorigenesis, and genomic integrity. Sci China Life Sci.

[22] Huang CJ, Chien CC, Yang SH (2008). Faecal ribosomal protein L19 is a genetic prognostic factor for survival in colorectal cancer. J Cell Mol Med.

[23] Huang CJ, Yang SH, Lee CL (2013). Ribosomal protein s27-like in colorectal cancer: a candidate for predicting prognoses. PLoS One.

[24] Iyer DN, Sin WY, Ng L (2019). Linking stemness with colorectal cancer initiation, progression, and therapy. World J Stem Cells.

[25] Neal CP, Fry AM, Moreman C (2014). Overexpression of the Nek2 kinase in colorectal cancer correlates with beta-catenin relocalization and shortened cancer-specific survival. J Surg Oncol.

[26] Takahashi Y, Iwaya T, Sawada G (2014). Up-regulation of NEK2 by microRNA-128 methylation is associated with poor prognosis in colorectal cancer. Ann Surg Oncol.

