# Supplementary material for: RPL17 Promotes Colorectal Cancer Proliferation and Stemness through ERK and NEK2/β-catenin Signaling Pathways
Source: J Cancer. 2022 May 13;13(8):2570–83. doi: 10.7150/jca.69428 (PMC9174872; doi:10.7150/jca.69428)
Supplement: Supplementary file 1 — Supplementary figure. [file jcav13p2570s1.pdf]

Supplementary Figure 1

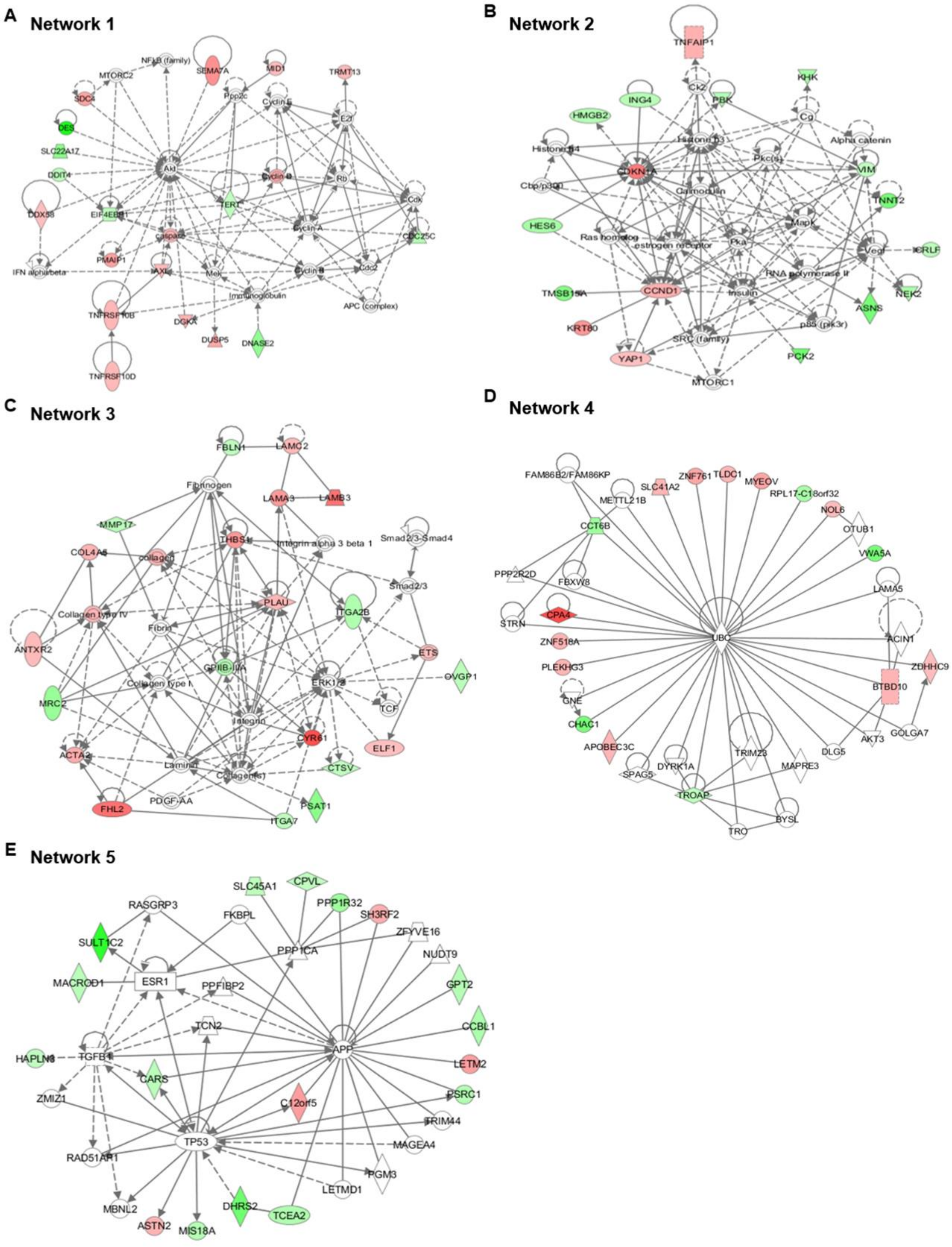

Figure S1. Functional connectivity of 159 commonly dysregulated genes in HCT119 and HT29 cells treated with RPL17siRNA for 48 h. (A-E), Five putative IPA networks with high score (>19). Up- and down-regulated genes are shown in red and green, respectively. Genes in gray are associated with the regulated genes.
